# Path optimization of taxi carpooling

**DOI:** 10.1371/journal.pone.0203221

**Published:** 2018-08-30

**Authors:** Changxi Ma, Ruichun He, Wei Zhang

**Affiliations:** School of Traffic and Transportation, Lanzhou Jiaotong University, Lanzhou, China; Beihang University, CHINA

## Abstract

The problem that passengers are hard to take taxis while empty driving rate is high widely exists under the traditional taxi operation mode. The implementation of taxi carpooling mode can alleviate the problem in a certain extent. The objective of this study is to optimize the taxi carpooling path. Firstly, the taxi carpooling path optimization model with single objective and its extended model with multiple objectives are built respectively. Then, the single objective path optimization model of taxi carpooling is solved based on the improved single objective genetic algorithm, and the multiple-objective path optimization model of taxi carpooling is solved based on the improved multiple-objective genetic algorithm. Finally, a case study is carried out based on a road network with 24 nodes. The case study results show the path optimization models and algorithms of taxi carpooling proposed in the paper can quickly get the taxi carpooling path, and can increase the income of taxi driver while reduce the cost for passengers.

## 1. Introduction

Taxi is an important transport mode in urban transportation system. Encouraging passengers sharing taxies could increase the transportation efficiency, shorten the total distance and cutting off idling time, alleviate road congestion, and reduce emissions [1]. At present, many scholars have carried out researches on taxi carpooling problem. Ardekani et al. proposed the taxi carpooling rate decision method where driver wages, vehicle operating cost, and other practitioner’s reasonable compensation cost are shared by the travel time or travel distance on the basis of vehicle fuel consumption rate [2]. Morisugi et al. got demand function Jakarta Indonesia using micro economics method, considering quasi linear indirect utility function of cost rate and passenger carrying rate. The cost function is composed of fixed cost and variable cost, and then the linear regression method is used to estimate the parameters. The maximum of social welfare is taken as the goal to calculate the best taxi rate and scale [3]. Chang et al. established a stochastic optimal control model with flexible start price to keep reasonable empty driving rate and meet the market demand [4]. Zou et al. established the transportation network model to calculate the shortest carpooling path, and analyzed the how road factors can influence on the shortest path [5]. Zhou developed a model of taxi path selection as well as rate optimization based on the fairness principle, which takes passengers’ minimum travel time and cost as the objective function and guarantees drivers’ reasonable income as constraints, considering the interests of drivers and passengers. The genetic algorithm was used to solve the model [6]. Guo et al. constructed the distance matrix of road traffic network, and they solved the shortest distance and determined the shortest route based on the matrix iteration method in the operations research, and carried out analysis for a certain road network [7]. Wang discussed the static and dynamic carpooling modes, dividing carpooling mode into several types, such as one to one, one to many and many to many modes. He developed the path selection method and rate optimization model [8]. Cheng et al. studied the dynamic taxi carpooling problem of many to many modes with different types of taxis and different types of passengers. The benefits that gained from travelers and drivers were considered, the time window constraint was introduced into the coordination mechanism, and the taxi carpooling optimization model was established [9]. Yan proposed effective route scheduling model, and designed solving method to deal with taxi carpooling problem well [10]. Chen et al. studied carpooling application using a social community, considering travel cost reduction [11]. Galland et al. studied individual mobility behavior in carpooling based on multi-agent simulation method [12]. Manzini et al. designed a decision support system for solving carpooling problem which has applied researches results into the reality [13]. Kammerdiener et al. built a classification model based on multiple constraints, and they solved the problem of ride-sharing partners [14].

Through analysis of the above research results, the current researches on the path optimization of taxi carpooling do not consider the driver's income and passengers' satisfaction at the same time, which causes limitation of the optimization results [15–17]. This paper constructs a taxi path optimization model considering the two aspects, which takes minimize taxi traveling distance and time as the objective function, takes taxi detour distance, passenger satisfaction, passenger fees and taxi driver income as constraints, and then the improved genetic algorithm is developed to solve the model.

This paper is arranged as follows: the second section constructs the path optimization model of taxi carpooling, the third section designs solution algorithm, the fourth section is case study, and the last section is the research conclusion and prospect.

## 2. Taxi carpooling path optimization model

### 2.1 Hypothesis condition

1. Each taxi drive is independent to others.
2. Vehicle capacity of each taxi is 3 passengers.
3. In order to reduce the waiting time, the taxi first takes all the passengers and then goes to the corresponding destination.
4. Passengers are not allowed to transfer.
5. The driving speed of a taxi keeps unchanged under the condition of carpooling and single riding taxi.
6. The taxi driver chooses the shortest path under the condition of single riding taxi.

### 2.2 Establishing the objective function

Detour can increase the passengers’ travel time to a certain extent in carpooling, so this paper takes the minimum taxi travel distance as the objective function, and optimizes the taxi carpooling path. The objective function is as follows:

$$\min z_{1}=\sum_{k\in n}\sum_{i\in A}\sum_{j\in A}y_{ij}^{l}\delta_{k}^{l}d_{ij}$$ (1)

where *A* is the set of nodes in road network, *i*,*j* ∈ *A*, *i*,*j* is adjacent; *d*_*ij*_ is the distance from *i* to *j*; *k* ∈ *n*, *n* is the set of taxis; *l* ∈ *p*, *p* is the set of carpooling path, and $y_{ij}^{l}=\{\begin{array}{c} \begin{array}{cc} 1 & \mathrm{when}\;\mathrm{carpooling},\;ij\in l \end{array}\\ \begin{array}{cc} 0 & \mathrm{when}\;\mathrm{carpooling},\;ij\notin l \end{array} \end{array}$, $\delta_{k}^{l}=\{\begin{array}{c} \begin{array}{cc} 1 & if\;l\;\mathrm{is}\;\mathrm{the}\;\mathrm{carpooling}\;\mathrm{path}\;\mathrm{of}\;\mathrm{vehicle}\;k \end{array}\\ \begin{array}{cc} 0 & \mathrm{otherwise} \end{array} \end{array}$.

### 2.3 Constraint conditions

#### 2.3.1 Detour constraint conditions

Carpooling may increase some passengers’ detour distance. For the whole taxi system, the overall travel distances can’t exceed to that of the same demand of travel distances with single riding taxi.

$$\sum_{k}\sum_{l}\sum_{i}\sum_{j}y_{ij}^{l}\delta_{k}^{l}d_{ij}\leq \sum_{K}\sum_{L}\sum_{i}\sum_{j}Y_{ij}^{L}\delta_{K}^{L}d_{ij}$$ (2)

where *K* ∈ *N*, *N* is the set of taxis with single riding taxi; *L* ∈ *P*, *P* is the set of paths with single riding taxi, and $Y_{ij}^{L}=\{\begin{array}{c} \begin{array}{cc} 1 & \mathrm{when}\;\mathrm{single}\;\mathrm{riding}\;\mathrm{taxi},\;ij\in L \end{array}\\ \begin{array}{cc} 0 & \mathrm{when}\;\mathrm{single}\;\mathrm{riding}\;\mathrm{taxi},\;ij\notin L \end{array} \end{array}$, $\delta_{K}^{L}=\{\begin{array}{c} \begin{array}{cc} 1 & if\;L\;\mathrm{is}\;\mathrm{the}\;\mathrm{single}\;\mathrm{riding}\;\mathrm{path}\;\mathrm{of}\;\mathrm{vehicle}\;k \end{array}\\ \begin{array}{cc} 0 & \mathrm{otherwise} \end{array} \end{array}$.

#### 2.3.2 Passengers satisfaction constraint

Taxi carpooling may lead to detour, and increase travel time. Passengers have direct perception of riding time, so travel time is used to describe passengers' satisfaction of carpooling.

$$s_{m}\geq \lambda$$ (3)

$$s_{m}=\{\begin{array}{c} \begin{array}{cc} 1, & \begin{array}{cc} if & t_{m}\leq T_{m}\left(1+\alpha \right) \end{array} \end{array}\\ \begin{array}{cc} 1-\frac{t_{m}-T_{m}\left(1+\alpha \right)}{t_{m}}, & \begin{array}{cc} if & t_{m}>T_{m}\left(1+\alpha \right) \end{array} \end{array} \end{array}$$ (4)

where *s*_*m*_ is the satisfaction of passenger *m*; *t*_*m*_ is the travel time of passenger *m* with carpooling (min); *T*_*m*_ is the travel time of passenger *m* with single riding taxi (min); *α* is the passengers’ tolerance for increasing of travel time (%); *λ* is the lower limit of passengers’ satisfaction.

#### 2.3.3 Passenger carpooling cost constraint

The starting point and a destination of each passenger may be different. The cost of a journey is shared by the passengers on the taxi. So a reasonable cost sharing method is needed in order to reflect the principle of fairness. If a passenger's travel distance is within the mileage of the taxi starting price, he or she only shares the starting price with the other passengers.

$$r_{ij}^{m}=\frac{r_{0}}{x_{m}^{k}}$$ (5)

$$c_{m}=\{\begin{array}{c} \begin{array}{cc} \frac{C_{0}}{X_{m}^{k}}, & \begin{array}{cc} if & \sum_{i}\sum_{j}y_{ij}^{l}\delta_{k}^{l}\delta_{m}^{k}d_{ij}\leq d_{0} \end{array} \end{array}\\ \begin{array}{cc} \frac{C_{0}}{X_{m}^{k}}+\sum_{i}\sum_{j}r_{ij}^{m}y_{ij}^{l}\delta_{k}^{l}\delta_{m}^{k}d_{ij}, & \begin{array}{cc} if & \sum_{i}\sum_{j}y_{ij}^{l}\delta_{k}^{l}\delta_{m}^{k}d_{ij}>d_{0} \end{array} \end{array} \end{array}$$ (6)

$$C_{m}=\{\begin{array}{c} \begin{array}{ccc} C_{0}, & if & Y_{ij}^{L}\delta_{K}^{L}\delta_{m}^{K}d_{ij}\leq d_{0} \end{array}\\ \begin{array}{ccc} r_{0}(Y_{ij}^{L}\delta_{K}^{L}\delta_{m}^{k}d_{ij}-d_{0})+C_{0}, & if & Y_{ij}^{L}\delta_{K}^{L}\delta_{m}^{K}d_{ij}>d_{0} \end{array} \end{array}$$ (7)

Such carpooling as the situation in which each passenger’s cost of carpooling is less than that of riding alone paid by the passengers.

$$c_{m}\leq C_{m},\forall m$$ (8)

where $X_{m}^{k}$ is the maximum number of passengers sharing with passenger *m* in vehicle *k*; $x_{m}^{k}$ is the number of passengers with passenger *m* on the road section *ij* of vehicle *k*; $r_{ij}^{m}$ is the unit mileage cost shared of passenger *m* on the road section *ij* (CNY/km); *c*_*m*_ is the cost of passenger *m* with carpooling. *C*_*m*_ is the cost of passenger *m* riding lonely. *d*_0_ is start mileage (km); *C*_0_ is starting price(CNY); *r*_0_ is price per kilometer(CNY/km), $\delta_{m}^{k}=\{\begin{array}{c} \begin{array}{cc} 1 & \mathrm{passenger}\;m\;\mathrm{rides}\;\mathrm{vehicle}\;k \end{array}\\ \begin{array}{cc} 0 & \mathrm{else} \end{array} \end{array}$.

#### 2.3.4 Driver income constraint

In order to protect the interests of taxi drivers, income of the driver with carpooling should be higher than the cost of the passenger whose travel distance is the longest distance among passengers with single riding taxi.

$$r_{ij}^{m}\sum_{m}\sum_{i}\sum_{j}y_{ij}^{l}\delta_{k}^{l}\delta_{m}^{k}d_{ij}\geq \max\;\left\{C_{m}\delta_{m}^{k},\forall m\right\},\forall k$$ (9)

#### 2.3.5 Model expansion

In the above modeling process, it is assumed that all taxis are traveling at a constant speed. However, as urban traffic demand increases, traffic congestion often occurs, so the speed of taxis cannot be maintained at a constant speed. In this way, we can further expand the model.

Assuming that the taxi speed is not uniform, for the optimization problem of taxi carpooling path, we need to add an objective function based on the aforementioned optimization model. This paper takes the minimum taxi travel time as the objective function, and optimizes the taxi carpooling path. The objective function is as follows:

$$\min z_{2}=\sum_{k\in n}\sum_{i\in A}\sum_{j\in A}y_{ij}^{l}\delta_{k}^{l}c_{ij}$$ (10)

where *c*_*ij*_ is the distance from *i* to *j*.

## 3. Algorithm design

The problem of taxi carpooling is a Non-deterministic Polynomial complete problem [9], therefore, the traditional simple algorithm cannot be used to solve the model effectively. Genetic algorithm can avoid the constraints of problem features, such as linearity, continuity, differentiability, and multimodality. Several feasible results of the optimization problem can be acquired by parallel operation on one chromosome, which is an effective algorithm for large-scale combinational optimization problems. Therefore, the genetic algorithm is introduced to solve the problem of taxi carpooling.

### 3.1 Single objective genetic algorithm

Before the model expands, the optimization model of taxi carpooling is a single objective optimization model, and the single objective genetic algorithm is used to solve it.

#### 3.1.1 Encoding

In order to ensure the passengers’ comfort with carpooling, the taxi is allowed to carry three passengers. The passengers will be randomly numbered. The passengers with the same starting position and destination will be compiled into the same number, which is called a group. The taxis will carry passengers in code order. If there are *N*_*pas*_ passengers in the road network, encoding length is *L*_*code*_ based on natural number encoding.

$$L_{code}=\{\begin{array}{cc} N_{pas} & \mathrm{the}\;\mathrm{starting}\;\mathrm{position}\;\mathrm{and}\;\mathrm{destination}\;\mathrm{are}\;\mathrm{the}\;\mathrm{same}\\ N_{pas}-\sum_{i=1}^{G_{p}}n_{i}+i & \begin{array}{l} \mathrm{the}\;\mathrm{starting}\;\mathrm{position}\;\mathrm{and}\;\mathrm{destination}\;\mathrm{of}\;n_{i}\;\mathrm{passengers}\\ \mathrm{of}\;\mathrm{group}\;i\;\mathrm{are}\;\mathrm{the}\;\mathrm{same} \end{array} \end{array}$$ (11)

where *G*_*p*_ is the number of groups with the same starting position and destination.

So the number of taxi *N*_*pas*_ passengers needs is

$$N_{taxi}=\{\begin{array}{cc} \frac{N_{pas}}{3} & N_{pas}\mathrm{is}\;\mathrm{multiple}\;\mathrm{of}\;3\\ \left[\frac{N_{pas}}{3}\right]+1 & \mathrm{else} \end{array}$$ (12)

**Fig 1:**
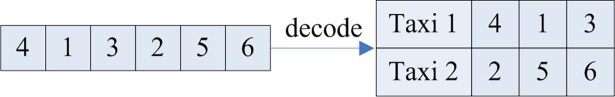
Encoding 1.

If six passengers want to take the taxi (the starting position and destination are not exactly the same), the encoding process is showed in Fig 1.

**Fig 2:**
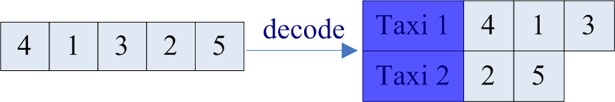
Encoding 2.

If the starting position and destination of the passenger mentioned above are the same, the encoding process is showed in Fig 2.

#### 3.1.2 Fitness function

Fitness function has a close relationship to the objective function. Fitness function of this algorithm is as follows:

$$F(i)=\{\begin{array}{cc} C_{\max}-z_{1}(i) & \mathrm{if}\;C_{\max}-z_{1}(i)>0\\ 0 & \mathrm{if}\;C_{\max}-z_{1}(i)\leq 0 \end{array}$$ (13)

where *z*_1_(*i*) is the objective function value of the model, *C*_max_ is a larger number.

#### 3.1.3 Selection operation

The good chromosomes are expected to be inherited with higher probability, and the inferior chromosomes enter the next generation with lower probability. Firstly, the population is sorted, and the probability of chromosome inheritance to the next generation is determined by the evaluation function. Then, roulette method is used to select operation. Evaluation function based on the order is used in this paper. The individuals are sorted in descending order based on *F*(*i*), and choice is carried out according to the following ways[18–19].

$$F\left(i\right)=\overset{n}{\underset{i=1}{\Pi }}a,0<a<1$$ (14)

$$E\left(i\right)=\frac{F\left(i\right)}{\sum_{j=1}^{n}F\left(j\right)}$$ (15)

It is obvious that *F*(*i*) decreases with *i* increases, and the gap between *F*(*i*) can be changed through updating the *a* value.

$$q_{0}=0$$ (16)

$$q_{i}=\sum_{j=1}^{i}E\left(i\right)$$ (17)

The cumulative probability of chromosome entry into the next generation is determined by the evaluation function. A random number between 0 and 1 is generated. And if *r* meets *q*_*i*−1_ ≤ *r* ≤ *q*_*i*_, the chromosome *i* is selected to enter the next generation.

#### 3.1.4 Crossover operation

**Fig 3:**
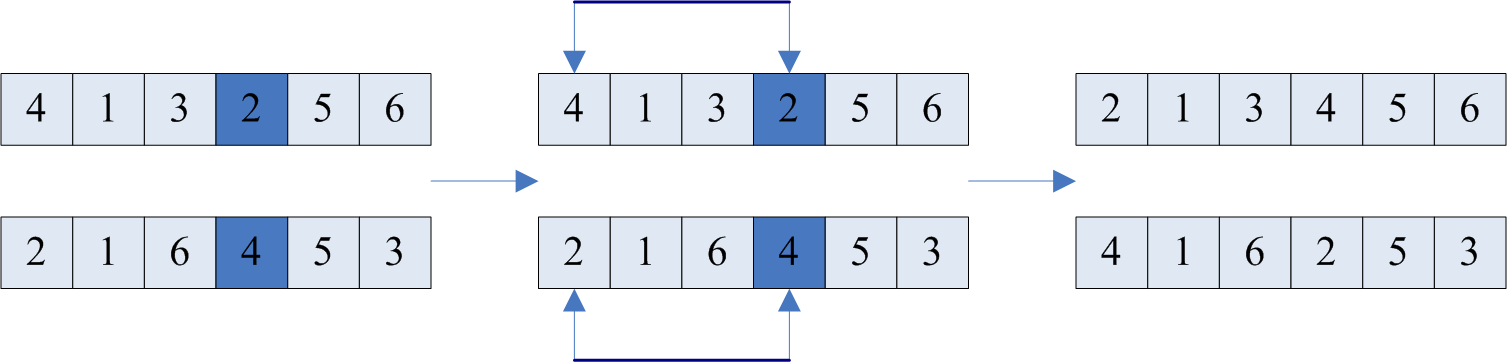
Crossover operation.

The crossover operation is the main step to generate new individuals of the generation. Cross probability *p*_*c*_ (0<*p*_*c*_<1) is given in this algorithm. A random number *r* ∈ (0,1) is generated. If *r* < *p*_*c*_, a bit is selected randomly, exchanges genetic, and each code finishes necessary internal exchange to prevent some passengers from being taken multiple times or not being taken, which avoid generating infeasible solutions. The rule is showed in Fig 3.

#### 3.1.5 Mutation operation

**Fig 4:**
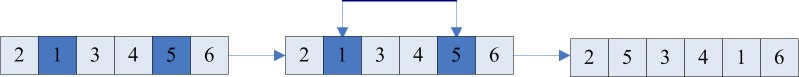
Mutation operation.

Mutation can improve the local search ability of genetic algorithm and the diversity of location groups [11]. Normally, mutation probability *p*_*m*_ (0<*p*_*m*_<1) is given, and a random number *r* ∈ (0,1) is generated. If *r* < *p*_*m*_, two different positions of the chromosome are determined, then exchange them. Mutation rule is showed in Fig 4.

#### 3.1.6 Termination rule

The number of the maximum evolutionary is used as the termination judgment condition, that is, when the cumulative number of generations in which the optimal solution does not change is more than a sufficiently large positive integer *N*, the algorithm terminates.

### 3.2 Multiple-objective genetic algorithm

#### 3.2.1 Algorithm design idea

The extended optimization model of taxi carpooling is a multiple-objective optimization model. For the multiple-objective optimization, the representative solution algorithms are Niched Pareto Genetic Algorithm (NPGA), Non Dominated Sorting Genetic Algorithm (NSGA-II), and Strength Pareto Evolutionary Algorithm (SPEA2), etc. [20].

Those algorithms all have favorable solving efficiency in specific issue. However, for a concrete issue, they cannot be applied directly but applied after revised according to the attribute of the issue [21–22]. This paper, aiming the characteristics of multiple-objective optimization of taxi carpooling path and combining the idea of multi-objective optimization, designs an improved genetic algorithm to solve the multiple-objective problem.

Different with single-objective optimization, the multiple objectives in multi-objective optimization can conflict with each other, and the improvement of one sub-objective may lower the performance of another sub-objective [23]. In other words, it is impossible to make multiple sub-objectives reach optimal. Therefore, the multi-objective optimization will generally get a non-inferior solution set. The elements of the set are called Pareto optimal solutions or non-inferior optimal solution. The Pareto optimal solution means there is no better solution superior to one of the objectives and not inferior to other objectives. In other words, it is impossible to optimize some objectives without deteriorating other objectives. For all objectives, the elements in Pareto optimal solution set are incomparable with each other. For actual application, one or multiple solutions from the Pareto optimal solution set of the multi-objective problem according to the understanding of the problem and preference of decision makers as the optimal solution of the multi-objective optimization problem to be solved. Therefore, the main task to solve the multi-objective optimization problem is to determine as more as and extensively distributed Pareto optimal solutions.

The genetic algorithm is a group operation algorithm. Through concurrent operation of one population, the multiple feasible solutions of the optimal problem can be found out without being restricted by property of the problem. The multi-objective optimization problem is mainly to find out a Pareto optimal solution set. Therefore, the genetic algorithm is an effective mean in Pareto optimal solution set to solve multi-objective optimization problem.

**Fig 5:**
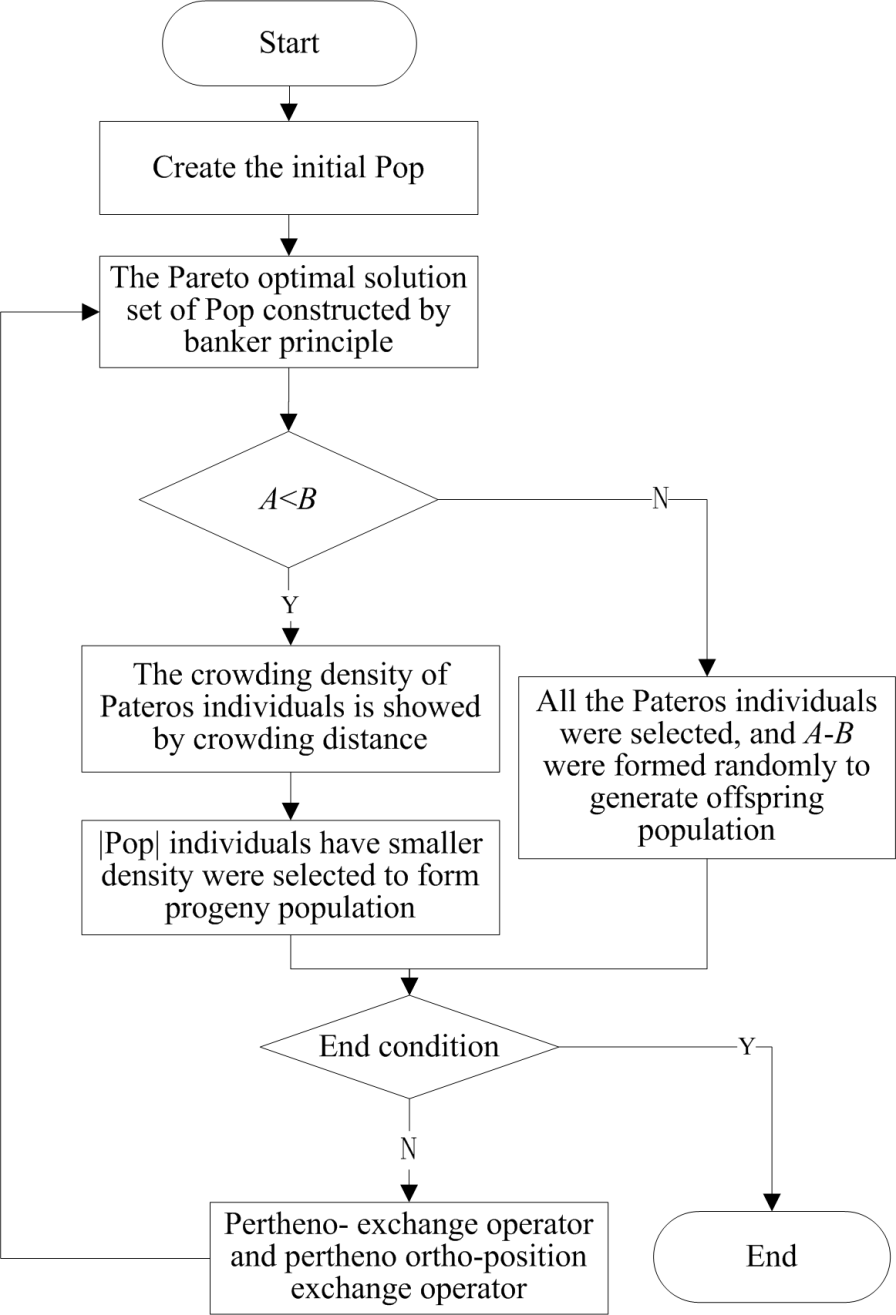
Flow diagram of the algorithm.

This paper, according to the framework of common multi-objective evolutionary algorithm, designs an improved genetic algorithm to solve the multiple-objective optimization problem of taxi carpooling path. This algorithm adopts banker principle to construct the Pareto optimal solution set, and keeps the distributivity of improved groups by crowding density method. In the aspect of genetic manipulation, this algorithm abandons the traditional crossover and mutation operators, and designs a kind of pertheno-genetic algorithm according to the characteristics of taxi carpooling problem. The flow diagram of the algorithm is showed in Fig 5, in which, Pop is population and |pop| is the scale of population, which is expressed by *A*; Paretos is the Pareto optimal solution set, and |Paretos| is number of the Pareto optimal solutions in the solution set, which is expressed by *B*. The end conditions adopt certain algebra constraints.

#### 3.2.2 Genetic operator design

The selection operators, crossover operators and mutation operators are important parts in genetic algorithm. Their design decides the solving efficiency of genetic algorithm. The specific operation of operator selection can be seen in Fig 5. The crossover operators and mutation operators are easy to generate large amount of infeasible solutions. Therefore, this paper, combining the characteristics of taxi carpooling problem, designs a pertheno-exchange operator and pertheno ortho-position exchange operator to replace the traditional crossover and mutation operators.

1. Pertheno-exchange operator: select two different gene positions of parental chromosome at random, and exchange the positions of the two genes to form filial chromosome. Apparently, the exchange will not generate infeasible solution.
2. Pertheno ortho-position exchange operator: select two different gene positions of parental chromosome at random, and confirm the staring and end position of ortho-position exchange section according to the sequence of the two gene positions. If the genes within the starting and end position are of even number, exchange all odd number genes with the odd even number genes to the right; if the genes within the starting and end position are of odd number, the last odd number gene remains unchanged while all other odd number genes are exchanged with the even number genes to the right. Apparently, the ortho-position exchange will not generate infeasible solution, either.

#### 3.2.3 Pareto optimal solution set constructed by banker principle

The banker principle is a kind of non-backtracking method. The newly constructed Pareto optimal solution needs not to compare with the existing Pareto optimal solutions. Supposed Paretos is the Pareto optimal solution set, and Pop is improved population, the Pareto optimal solution set of Pop constructed by banker principle is as follows:

Step1: initialize Pareto optimal solution set.
Srep2: take an individual *a* (generally the first individual) from Pop as the banker, and compare the banker with other individuals in sequence. When comparing, the individuals dominated by banker will be deleted.
Step3: after compared the banker with all individuals in Pop, if an individual *b* dominates banker *a*, the banker *a* will be deleted. Otherwise, the banker *a* will be put into Paretos.
Step4: repeat step2 and step3 till Pop is empty.

#### 3.2.4 Individual crowding density calculation

The distributivity of improved population is an important part of multiple-objective optimization. The crowding density method is adopted to keep the distributivity of the improved population. Specifically, the crowding distance is used to show the crowding density of individuals. The larger the crowding distance of an individual is, the smaller crowding density is. Before calculating the crowding distance of each individual, sort the population according to the each sub-objective function value. Supposed DistancePop[*i*] is the crowding distance of individual *i*, and Pop[*i*]. f[*j*] is the *j*^th^ objective value of individual *i*, and if there are *m* objectives, the crowding distance of individual *i* will be:

$$\mathrm{DistancePop}\left[i\right]=\sum_{k=1}^{m}\left(\mathrm{Pop}[i+1].f[k]-\mathrm{Pop}[i-1].f[k]\right)$$ (18)

## 4. Case study

**Fig 6:**
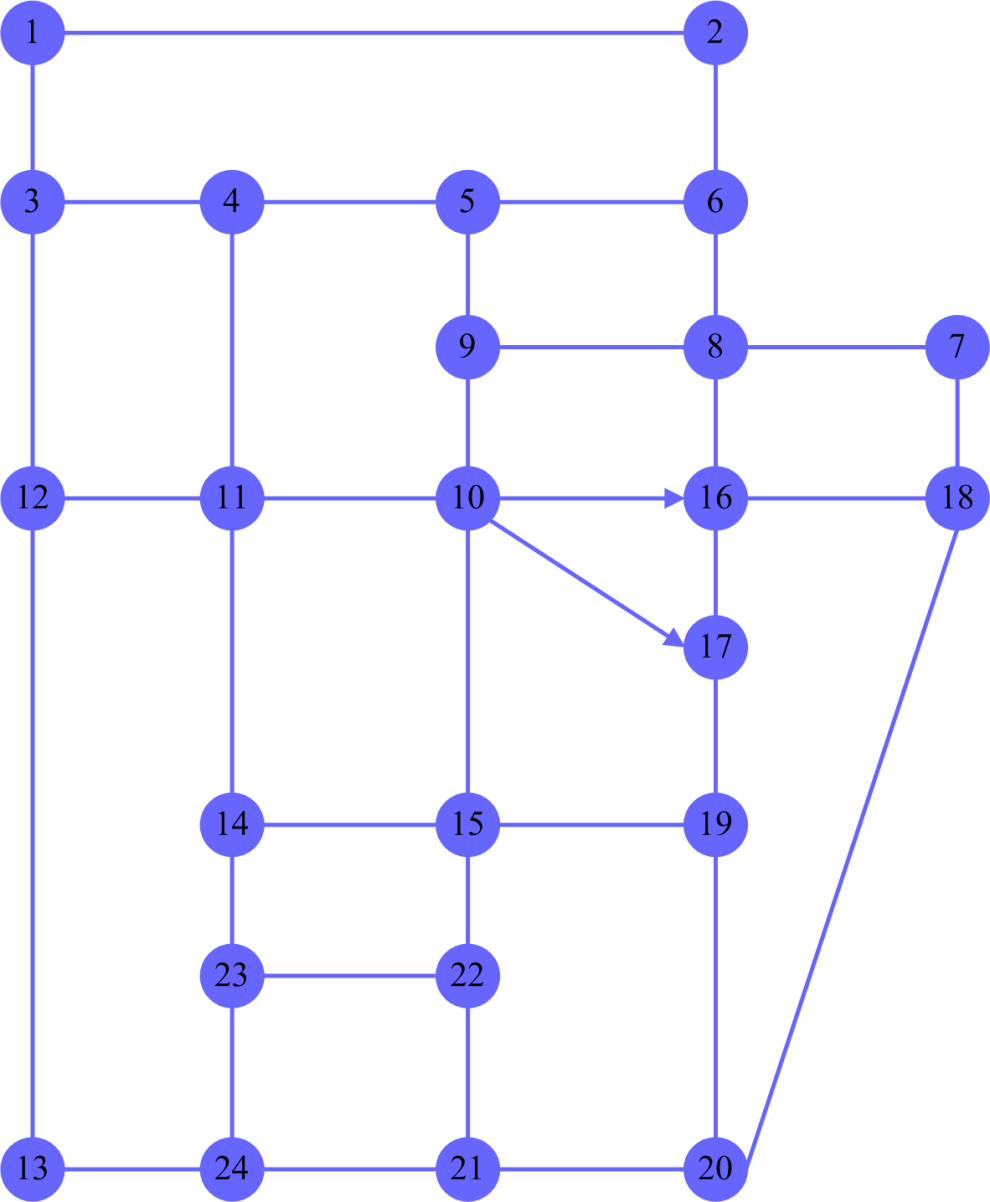
A road network with 24 nodes.

Suppose there are nine passengers and they want to take taxis in a road network with 24 nodes(Fig 6). The distance between adjacent nodes is a random value between 1km and 8km. The traveling time between adjacent nodes is a random value between 0.2h and 4h. Three taxis are needed for the nine passengers. The nine passengers are numbered randomly as in Table 1. The single objective genetic algorithm of taxi path optimization is achieved with Visual Studio6.0. Parameters are set as follows: PopSize = 200, Gen = 1000, *P*_*c*_ = 0.1, *P*_*m*_ = 0.1, *V* = 50km/h, *d*_0_ = 3km, *C*_0_ = 10CNY; *r*_0_ = 1.4CNY/km, *α* = 0.4, *λ* = 0.6, *a* = 0.9.

**Table 1:** Passengers information.

Passenger No.	The number of passengers	Demand point
1	1	1–20
2	1	2–24
3	1	21–9
4	1	3–17
5	1	6–14
6	1	22–4
7	1	5–16
8	1	10–18
9	1	19–1

**Fig 7:**
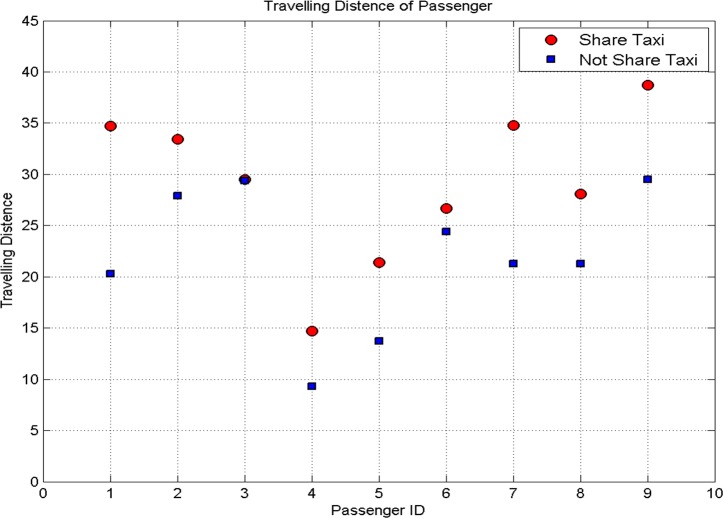
Travel mileages of passengers.

**Fig 8:**
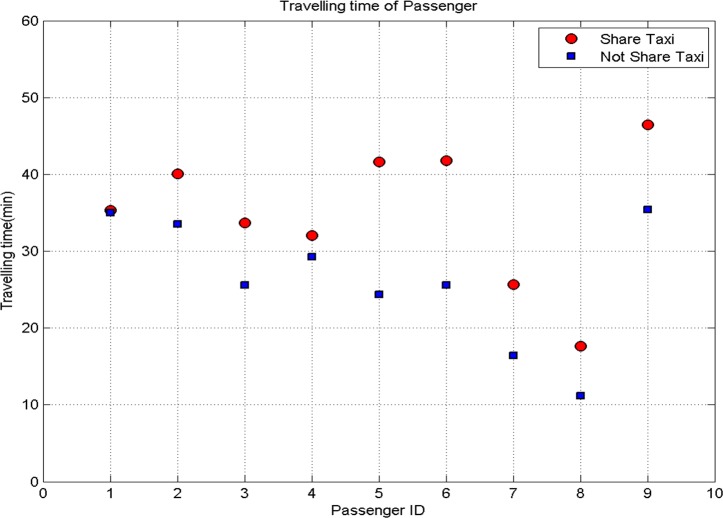
Travel time of passengers.

The total travel distance and travel time of passengers with carpooling and non-carpooling are showed in Fig 7 and Fig 8 respectively.

The income of taxi driver, travel mileages and carpooling path with carpooling and non- carpooling are showed in Table 2.

**Table 2:** Taxi income.

Taxi No.	Carpooling passenger	Carpooling income /(CNY)	Non-carpooling income /(CNY)	Carpooling mileage /(km)	Non-carpooling mileage /(km)	Carpooling path
1	5,2,1	69.16	46.96	49.4	77.6	1-2-6-8-7-18-20-21-24-23-14
2	8,7,4	46.75	39.96	33.4	47.4	3-4-5-9-10-17-16-18
3	6,3,9	67.35	47.1	44.1	72.1	21-22-15-19-15-10-9-5-4-3-1
Total		183.26	134.02	126.9	197.1	-

Note: Non-carpooling refers to the income driver get when he only takes the passengers whose travel mileage is the largest among the carpooling passengers; Non-carpooling mileage refers to the total mileage meeting the three passengers with non-carpooling.

**Fig 9:**
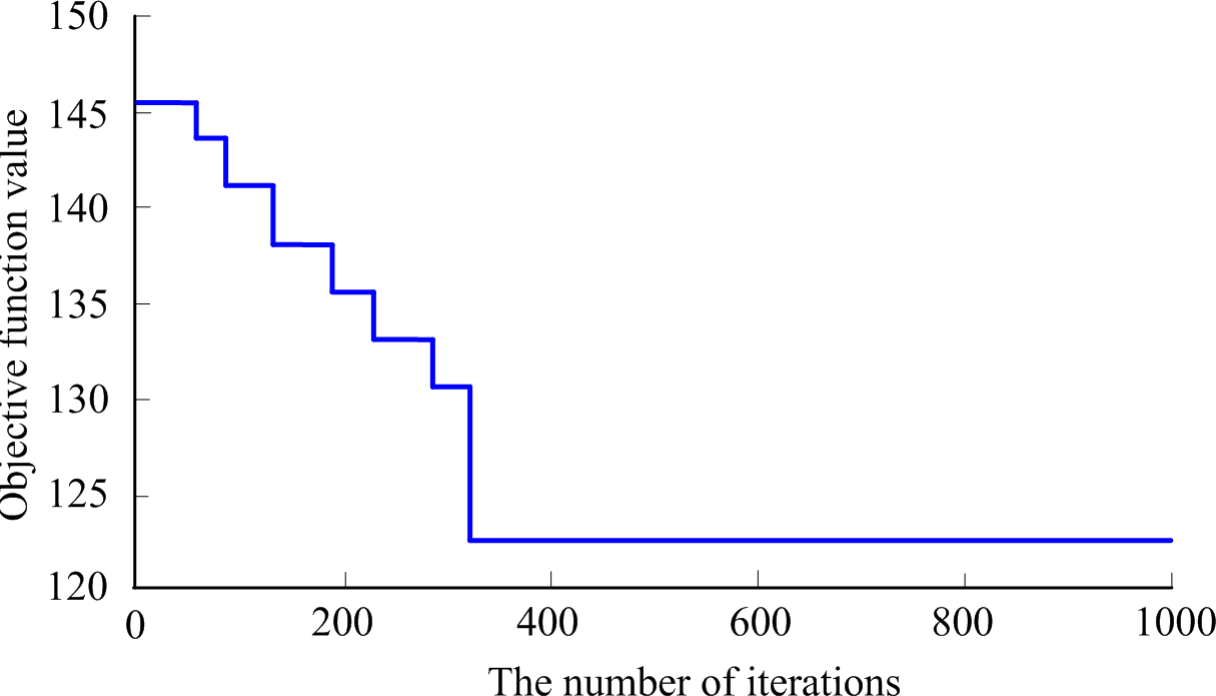
The GA evolution curve.

The single objective genetic algorithm evolutionary graph for solving the carpooling path optimization is showed in Fig 9.

The above calculation results show that the total mileages of three taxis are 126.9km with carpooling, which shortens about 35.6% compared with non-carpooling. By comparison with the cost for the passenger whose travel mileage is the largest with non-carpooling, the increasing values of the three taxis are 22.2 CNY, 6.8 CNY and 20.24 CNY respectively, and the taxi income is increased by about 36.7%. The nine passengers could save up to144.88 CNY in total.

For the extended model, we have written an improved multi-objective genetic algorithm program based on Visual Studio6.0. We set the size of population PopSize = 200, Evolutionary generation MaxGen = 200, Crossover rate CrossRate = 0.95, and Mutation rate MutationRate = 0.1. Other parameters have the same values as before. The Pareto solution set can be obtained by calculation, which are showed in Table 3.

**Table 3:** Pareto solution.

Pareto solution	Taxi No.	Carpooling path
Pareto 1	1	1-2-6-8-16-18-20-21-24-23-14
	2	3-4-5-9-10-16-17-16-18
	3	21-22-15-19-17-16-8-9-5-4-3-1
Pareto 2	1	1-2-6-8-7-18-20-21-24-23-14
	2	3-4-5-9-10-17-16-18
	3	21-22-15-19-15-10-9-5-4-3-1
Pareto 3	1	1-2-6-8-7-18-20-21-24-23-14
	2	3-4-5-9-10-16-17-16-18
	3	21-22-15-19-17-16-8-9-5-4-3-1
Pareto 4	1	1-2-6-8-16-18-20-21-24-23-14
	2	3-4-5-9-10-17-16-18
	3	21-22-15-19-15-10-9-5-4-3-1
Pareto 5	1	1-2-6-8-16-18-20-21-24-23-14
	2	3-4-5-9-10-17-16-18
	3	21-22-15-19-17-16-8-9-5-4-3-1
Pareto 6	1	1-2-6-8-7-18-20-21-24-23-14
	2	3-4-5-9-10-16-17-16-18
	3	21-22-15-19-15-10-9-5-4-3-1
Pareto 7	1	1-2-6-8-16-18-20-21-24-23-14
	2	3-4-5-9-10-16-17-16-18
	3	21-22-15-19-15-10-9-5-4-3-1
Pareto 8	1	1-2-6-8-7-18-20-21-24-23-14
	2	3-4-5-9-10-17-16-18
	3	21-22-15-19-17-16-8-9-5-4-3-1

The strength Pareto genetic algorithm (SPEA) is used to test the efficiency of the improved multi-objective genetic algorithm. The algorithm parameter and Pareto optimal solution set selection strategy are the same as those of the improved multi-objective genetic algorithm designed in this paper. The results are showed in Table 4.

**Table 4:** Performance comparison between SPEA and improved multi-objective genetic algorithm.

Optimization objective	SPEA	Improved multi-objective genetic algorithm
Mean value of distance objective/(km)	128.23	127.71
Mean value of time objective/(h)	3.24	2.85
Run time/(s)	25	19

From Table 4, we can see the mean values of two objective functions obtained from the improved multi-objective genetic algorithm designed in this paper are better than SPEA, and the run time is reduced. The results show that the improved multi-objective genetic algorithm designed in this paper can not only obtain a more satisfactory solution, but also has faster convergence speed compared with the traditional genetic algorithm.

## 5. Research conclusions and prospects

The taxi carpooling system is researched in this paper, and the following conclusions are obtained.

(1) The path optimization model of taxi carpooling system is built with taxi travel distance minimum as the objective function, and detour distance, passenger satisfaction, cost of passengers and taxi driver income as constraints. The genetic algorithm is used to solve the model, and the case study results show that the optimal model and the algorithm are feasible. For the model, algorithm and program code has important reference value for the development and perfecting function of taxi APP.

(2) The case study shows the passenger carpooling shortens the total travel distance of taxi under the condition of the same travel demand. Carpooling can contribute to the mitigation of urban traffic congestion and energy saving and emission reduction to some extent. Although the carpool will make some passengers detour distance and increase the travel time, but the operation mode can greatly reduce passengers’ cost.

(3) Taxi carpooling can increase drivers’ income and reduce the passengers’ cost by the above analysis, which can make the driver and passengers to reach a "win-win" situation, and alleviates travel difficult problem as well.

After the research on the taxi carpooling path optimization model and algorithm, the development of carpooling APP and the design of scientific cost sharing meter are key content of the next research stage.
